# Copy number variations of cytochrome *P450* genes in Kinh Vietnamese

**DOI:** 10.2478/abm-2023-0048

**Published:** 2023-09-17

**Authors:** Nhung Phuong Vu, Ton Dang Nguyen, Binh Huy Nguyen, Duong Thuy Nguyen, Hai Van Nong, Ha Hai Nguyen

**Affiliations:** Department of Biotechnology, Graduate University of Science and Technology, Vietnam Academy of Science and Technology, Hanoi 100000, Vietnam; Genome Analysis Laboratory, Institute of Genome Research, Vietnam Academy of Science and Technology, Hanoi 100000, Vietnam; Department of Physiology, Hanoi Medical University, Dong Da, Hanoi 100000, Vietnam

**Keywords:** CNVs, *CYP450*, drug metabolism, genetic variants, pharmacogenetics

## Abstract

**Background:**

The cytochrome P450 (*CYP450*) family is well known as a major group of drug metabolizing enzymes. The polymorphism of *CYP450* genes is the main factor having an impact on the interindividual difference in drug response, including drug efficacy and drug safety. The single nucleotide polymorphism (SNPs) of Vietnamese Kinh has been widely studied, but information about the copy number variations (CNVs) of other *CYP450* genes is still unknown.

**Objective:**

To identify the CNV variability of *CYP450* in 154 healthy unrelated Kinh Vietnamese, except e*CYP2D6*, which was previously reported.

**Methods:**

Multiplex Ligation-Dependent Probe Amplification (MLPA) was applied for determination of copy number of 10 *CYP450* genes. Later, PCR or quantitative PCR (qPCR) was used to confirm the detected CNVs in randomly chosen subjects.

**Results:**

Of the 154 subjects, along with *CYP2D6*, 4 other *CYP450* genes showed CNVs including duplications (*CYP1B1*), deletions (*CYP2A6* and *CYP2C9*), and both duplications and deletions (*CYP2E1*). Among these, *CYP2A6* exhibited the greatest frequency of CNVs compared with other *CYP450*, in which *CYP2A6*Del accounted for 11%. Meanwhile, allele *CYP2E1*Del showed the lowest frequency with only 0.3%.

**Conclusions:**

The present study provides new insight into *CYP450* CNVs in the Kinh Vietnamese cohort. Our data have contributed to genetic profiling of *CYP450* CNVs in Vietnam, which would be helpful for facilitating implementation of pharmacogenetics in drug dosing adjustment in Vietnam.

Interindividual variability in drug response can lead to alteration of the therapeutic effect, which includes lacking drug efficacy or drug toxicity. The human cytochrome P450 (*CYP450*) enzyme superfamily is responsible for the oxidative metabolism of many drugs, xenobiotics, as well as other endogenous substrates. Inherited genetic variations in *CYP450* genes are well known as factors contributing to the difference in drug response among individuals. In recent years, the increase in genetic tests allowed detection of large numbers of human genetic variants. Despite single nucleotide polymorphisms (SNPs) of *CYP450* that were intensively studied among various populations worldwide, there is little information available about the copy number variations (CNVs) of these pharmacogenes.

CNV is a type of structural variation defined as duplications or deletions of DNA fragments ranging from 1 kb to 3 Mb, in which the number of copies of a particular gene differs from one individual to another. The completion of the Human Genome Project had made it clear that many genetic regions in the human genome experience gains and losses of genetic materials, which carry more or less than 2 copies. Until now, alleles that consisted of 0–13 gene copies had been described across the human population globally. Pharmacogenetic CNV alleles could play an important role in enzyme activity as well as drug response diversity. In fact, several works have described CNVs in pharmacogenes such as *CYP2B6*, *CYP2D6*, *GSTT1*, *GSTM1*, *SULTA1* and *SULTA2* [1,2,3]. In 2018, available CNVs data were investigated and revealed that several populations harbor CNV alleles at *CYP2C* gene locus, in which *CYP2C19* showed the highest number of novel deletions [4]. Recently, a report on 340 genes involved in absorption, distribution, metabolism, and excretion of drugs had identified 445 deletions and 167 duplications in 36 pharmacogenes, including the well-known CNVs of *CYP450* (*CYP2D6*, *CYP2A6*) [5].

The CNV data of *CYP450* and other pharmacogenes across populations still remain limited while the CNV profile of these genes in Vietnam had not been reported yet. Therefore, this study aimed to determine the prevalence of *CYP450* CNVs in Vietnamese Kinh—the largest ethnic group accounting for approximately 86% of the whole population. For copy number detection, multiplex ligation-dependent probe amplification (MLPA) was used and subsequently, quantitative PCR (qPCR) copy number assays or genotyping was performed to validate the observed CNVs. This study would provide new insights into *CYP450* CNVs frequency in a Vietnamese Kinh cohort, which extends the understanding of these CNVs in Asia and the potential application of data in translation of pharmacogenetics from the bench to bedside.

## Materials and methods

### Study subjects and DNA extraction

A total of 154 unrelated healthy Kinh volunteers (93 females and 61 males) from Hanoi Medical University of Vietnam were recruited. All subjects were regarded as healthy according to their medical history and physical examination. The ethnic identity of volunteers was identified based on their personal documents with at least 3 generation of corresponding parental ancestry. The study purpose was explained to all individuals and written informed consent was obtained from each subject before sample collection. This project was approved by the Institutional Review Board (IRB) of the Institute of Genome Research, Vietnam Academy of Science and Technology. The study is in accordance with the STROBE statement [6]. For all subjects, 2 mL of peripheral blood was collected and preserved in EDTA containing tubes. Genomic DNA was subsequently extracted from the blood samples using E.Z.N.A Blood DNA Mini Kit (USA) according to manufacturer's instructions.

### Multiplex ligation-dependent probe amplification

To identify deletions/duplications on *CYP450* gene, MPLA was performed using the commercial SALSA MLPA P128-C1 Cytochrome P450 Probemix kit (MRC-Holland, Amsterdam, Netherlands) following the manufacturer's instructions. The CNVs data of *CYP2D6* detected in these 154 subjects were separately reported [7]. In addition to *CYP2D6*, MLPA probemixs were specifically designed for 2–6 exons of other *CYP450* genes, including *CYP1A1*, *CYP1A2*, *CYP1B1*, *CYP2A6*, *CYP2B6*, *CYP2C9*, *CYP2C19*, *CYP2E1*, *CYP3A4,* and *CYP3A5*. Fifty nanograms of genomic DNA was denatured at 98°C for 5 min and cooled at 25°C. Denatured DNA were later hybridized with SALSA probemix at 60°C for 16–20 h. Subsequently, the annealed probes were ligated at 54°C for 15 min followed by heating at 98°C in 5 min. In the next step, complete ligation reactions were used for the PCR with thermocycle consisting of 35 cycles (95°C for 30 s, 60°C for 30 s and 72°C for 60 s), followed by incubation at 72°C for 20 min. The amplicons were separated by capillary gel electrophoresis and the collected data were analyzed using the Coffalyzer.net software. The copy number of subjects was determined by the final probe ratio distribution: 0 copy (0), 1 copy (0.4–0.65), 2 copies (0.8–1.2), 3 copies (1.3–1.65), and 4 copies (1.75–2.15).

### Copy number analyses of *CYP1B1, CYP2C9,* and *CYP2E1* by qPCR

Gene dosage of different samples was performed with relative quantification real-time PCR method. Real-time PCR reaction was performed using Luna Universal qPCR Master Mix (NEB) and RPPH1 (a reference gene with a single copy) primers used for quantitative analysis were referred to Ahani's study [8]. Additionally, primers were designed for specific detection of *CYP1B1* (exons 1 and 2), *CYP2E1* (exons 8 and 9) and *CYP2C9* (exons 4 and 7) (**Table 1**). The reaction was performed in 96 wells plate with 10 μL volume in total, which included 10 ng genomic DNA, 1X Luna Universal qPCR Mastermix (NEB), 0.25 μL for each primer (10 pmole/μL) and Ultrapure Distilled Water (ThermoFisher Scientific). Subsequently, the covered plates were run on LightCycler 96 Instrument (Roche) with the thermocycle with denaturation at 95°C for 10 min, following by 45 cycles (95°C for 15 s and 60°C for 60 s). The copy number of targeted exon in comparison to reference gene was determined according to the following equation: ΔΔCt = [CtRPPH1(Reference sample) − Ct targeted exon (reference sample)] − [CtRPPH1(Unknown sample) − Ct targeted exon (Unknown sample)]. The relative copy number of the genes was later calculated following the ratio equation (2^−ΔΔCt^).

**Table 1.** Primers sequence for real-time PCR detecting copy number of *CYP1B1*, *CYP2C9,* and *CYP2E1*

**Gene-region**	**Primer name**	**Sequence (5′-3′)**
*CYP1B1* Exon 1	CYP1B1 E1F	CTG CGACTCCAGTTGTGAGAG C
	CYP1B1 E1R	AGTCTCTTGGCGTCG TCAGTG
*CYP1B1* Exon 2	CYP1B1 E2F	CACAGCATGATGCGCAACTTC
	CYP1B1 E2R	CACTCATGACGTTGGCCA CG
*CYP2C9* Exon 4	CYP2C9 E4F	ATGCATGCCGAACTCTTTTT
	CYP2C9 E4R2	AGGATGAAAGTGGGATCACAGG
*CYP2C9* Exon 7	CYP2C9 E7F	CACATTTGTGCATCTGTAACCA
	CYP2C9 E7R3	CCGGTTTCTGCCAATCACACG
*CYP2E1* Exon 8	CYP2E1 E8F	GGCACAGTCGTAGTGCCAACTC
	CYP2E1 E8R	CTGCCTCTGATCTTTCTCACCTG
*CYP2E1* Exon 9	CYP2E1 E9F	TGGAGAAGGCCTGGCTCGCATG
	CYP2E1 E9R	GTTCAGGGTGTCCTCCACACAC

### *CYP2A6* genotyping

Five primers were used in 2-step PCR method in order to detect *CYP2A6* deletion allele as previously described by Oscarson et al. [9] (**Table 2**). Eight microliters of PCR II were aliquoted and subsequently analyzed on 1% agarose gel staining with ethidium bromide.

**Table 2.** Primer used for genotyping the *CYP2A6* deletion and duplication alleles [9]

**Primer name**	**Sequence (5′-3′)**
2A E7F	GRC CAA CAT GCC CTA CAT G
2A6R1	GCA CTT ATG TTT TGT GAG ACA TCA GAG ACA A
2A6E8F	CAC TTC CTG AAT GAG
2A7E8F	CAT TTC CTG GAT GAC
2A6R2	AAA ATG GGC ATG AAC GCC C

### Statistical analysis

Inter-ethnic comparison of genes copy number frequency between Kinh Vietnamese and global populations were assessed by Chi square (c^**2**^) and Fisher exact test. All statistical analyses were 2-sided and *P* < 0.05 was considered as statistically significant.

## Results

### Copy number profiling of *CYP450* genes in Vietnamese Kinh

**Figure 1.**
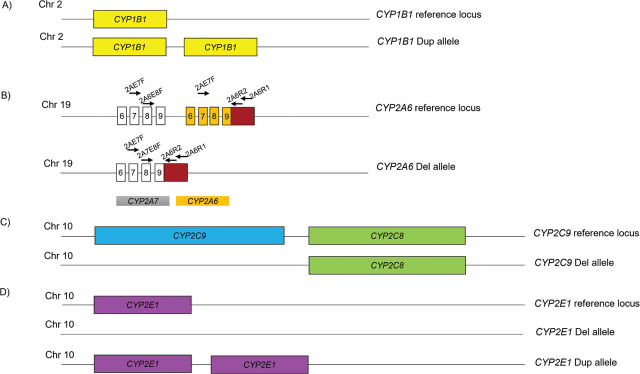
Graphical illustration of *CYP450* CNVs identified in current study. **(A)** Reference gen locus of *CYP1B1* (up) and *CYP1B1*Dup allele (down). *CYP1B1* located on chromosome 2 and represented by yellow boxes. **(B)** Reference gen locus on chromosome 19 of *CYP2A6* (orange box) and pseudogene *CYP2A7* (grey box) (up) and *CYP2A6*Del allele (down), downstream of exon 9 of *CYP2A6* is denoted by the black boxes. Primers position and strategy used for *CYP2A6* del allele detection are also shown. **(C)** Reference gene cluster on chromosome 10 including *CYP2C9* (blue box), *CYP2C8* (green box) (up), and *CYP2C9* Del allele (down). **(D)** Reference gen locus of *CYP2E1* (pink box) on chromosome 10 (up), *CYP2E1*Del allele (middle), and *CYP2E1*Dup allele with 2 copies of the gene (down). CNVs, copy number variations.

CNVs of *CYP450* genes were analyzed using MLPA with probes designed for 10 following genes except *CYP2D6*: *CYP1A1*, *CYP1A2*, *CYP1B1*, *CYP2A6*, *CYP2B6*, *CYP2C9*, *CYP2C19*, *CYP2D6*, *CYP2E1*, *CYP3A4,* and *CYP3A5*. Among 154 unrelated healthy individuals, CNVs were detected in only 4 genes including *CYP1B1*, *CYP2A6*, *CYP2C9* and *CYP2E1*. Of these genes, deletions were identified in *CYP2A6* and *CYP2C9*, duplications were found in *CYP1B1,* and only *CYP2E1* with both duplications and deletions was identified. The representative MLPA data from samples harboring variable copy numbers are shown in **Table 3**. Additionally, the detail of *CYP450* CNVs observed in studied subjects are shown in **Figure 1**.

**Table 3.** Final probe ratio from MLPA of representative samples carrying *CYP450* copy number alterations

**Samples**	***CYP1B1***		***CYP2A6***				***CYP2C9***					***CYP2E1***		
			
	**Exon 2**	**Exon 3**	**Exon 1**	**Exon 2**	**Exon 3**	**Exon 5**	**Exon 1**	**Exon 7**	**Exon 8-1**	**Exon 8-2**	**Exon 9**	**Exon 5**	**Exon 6**	**Exon 8**
FVN48	0.89	0.87	0.41	0.42	0.5	0.43	1.1	1.18	1.16	1.06	0.92	0.95	0.93	0.88
FVN74	0.89	0.85	0	0	0	0	1	1	1	0.96	0.99	1.02	0.94	1.11
MVN23	**1.34**	**1.53**	1.15	1.14	1.16	1.15	0.47	0.55	0.61	0.56	0.58	1.09	1.08	0.99
FVN73	1.08	0.99	1.05	1.07	1.04	1.08	1	0.99	0.98	0.92	0.96	0.51	0.51	0.47
FVN46	1.04	1.1	0.94	0.92	1.17	1.2	1.17	1.03	1.1	0.82	0.93	**1.37**	**1.38**	**1.63**
FVN98	0.86	0.86	0.97	0.98	1.16	0.91	1.06	0.96	1.02	1.28	1.11	**1.36**	**1.37**	**1.37**

Final probes ratio indicated deletions (underline); final probes ratio indicated duplications (bold).

MLPA, multiplex ligation-dependent probe amplification.

### Identification of *CYP1B1*, *CYP2A6*, *CYP2C9,* and *CYP2E1* CNVs

**Figure 2.**
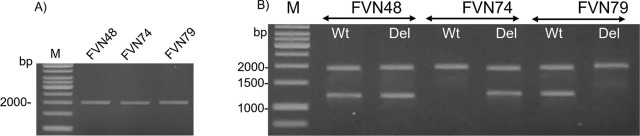
Copy number confirmation of *CYP2A6Del*. **(A)** Amplification of a part of *CYP2A6* gene or hybrid *CYP2A7*/*2A6* using primer pair *2AE7F/2A6R1* resulting in approximately 2 kb products. **(B)** Detection of *CYP2A6Del* allele, Wt: Wild-type allele, Del: Deletion allele. *CYP2A6* genotype of tested samples were as follows: FVN48: Wt/Del, FVN74: Del/Del, FVN79: Wt/Wt.

For *CYP2A6*, both homozygous and heterozygous deletions were detected in the studied subjects by MLPA with copy number probes designed for exons 1, 2, 3, and 5. Among 154 subjects, the most frequent genotype was Wt/Wt having 2 copies (80.52%), following by Wt/Del genotype having 1 copy (16,88%). The homozygous deletion of *CYP2A6* with Del/Del genotype having 0 copy made up only 2.6% (**Table 4**). The frequency of *CYP2A6* alleles ranged from 11% (*CYP2A-6*Del-0 copy) to 89% (*CYP2A6*Wt-1 copy) (**Table 5**). *CYP2A6* duplication alleles (2 copies) were not identified in this study. Samples showing deletion alleles of *CYP2A6* were randomly chosen for re-genotyping in order to confirm the MLPA results. The genotyping results were consistent with MLPA and indicated that deletions found in the studied subjects encompassed the entire *CYP2A6* gene (**Figure 2**).

**Table 4.** Genotypes frequency of *CYP450* copy number variability

**Genotype**	**Gene/genotypes frequency**							

	***CYP1B1***		***CYP2A6***		***CYP2C9***		***CYP2E1***	
			
	**N**	**Freq. (%)**	**N**	**Freq. (%)**	**N**	**Freq. (%)**	**N**	**Freq. (%)**
Wt/Del	0	0	26/154	16.88	3/154	1.95	1/154	0.65
Del/Del	0	0.00	4/154	2.60	0	0.00	0	0.00
Wt/Dup	3/154	1.95	0/154	0.00	0	0.00	3/154	1.95
Wt/Wt	151/154	98.05	124/154	80.52	151/154	98.05	150/54	97.40

**Table 5.** Alleles frequency of *CYP450* copy number variability

**Allele/copies**	**Gene/alleles frequency (n = 308)**							

	***CYP1B1***		***CYP2A6***		***CYP2C9***		***CYP2E1***	
			
	**Freq.**	**95% CI**	**Freq.**	**95% CI**	**Freq.**	**95% CI**	**Freq.**	**95% CI**
**Dup (2)**	0.010	0.000–0.010	-	-	-	-	0.010	0.000–0.021
**Wt (1)**	0.990	0.980–1.000	0.890	0.852–0.927	0.990	0.980–1.000	0.987	0.974–1.000
**Del (0)**	-	-	0.110	0.073–0.148	0.010	0.000–0.021	0.003	0.000–0.010

CI, confidence interval; n, number of alleles.

**Figure 3.**
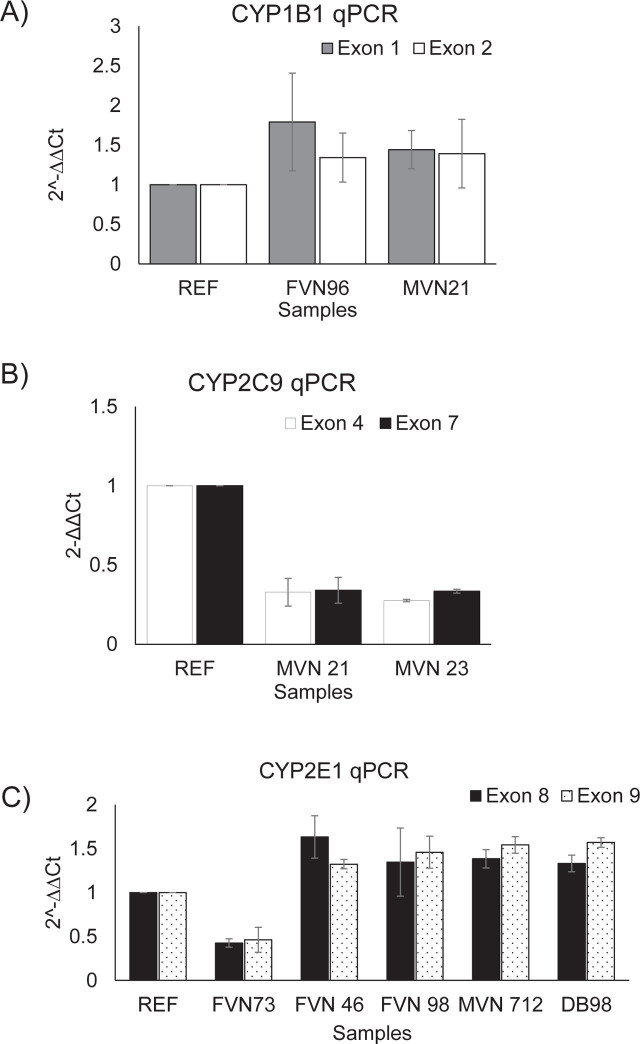
Copy number confirmation of *CYP1B1, CYP2C9*, and *CYP2E1*. Primers for real-time PCR located in exon 1 and exon 2 of *CYP1B1*, exons 4 and 7 of *CYP2C9*, exons 8 and 9 of *CYP2E1* were used to confirm CNVs detected by MLPA in these genes. REF: reference samples, in which MLPA showing 2 copies of gene of interest. **(A)** Heterozygous duplications of *CYP1B1* detected in both FVN 96 and MVN 21. **(B)** Heterozygous deletions of *CYP2C9* were found in both MVN 21 and MVN 23. **(C)** Copy number confirmation of *CYP2E1* showed that heterozygous deletion was detected in FVN 73 while heterozygous duplications were identified in FVN 46, FVN 98, Kinh 712 and DB98. CNVs, copy number variations; MLPA, multiplex ligation-dependent probe amplification.

For *CYP1B1*, MLPA data resulted in duplications in studied subjects with copy number probes specific for exons 1 and 3. Of 154 subjects, the homozygous wildtype was the most common genotype (98.05%). Meanwhile, heterozygous duplication of *CYP1B1* (Wt/Dup) made up only 1.95% (**Table 4**). There were 2 *CYP1B1* alleles identified with frequencies ranging from 1% (Dup-2 copies) to 99% (Wt-1 copy) (**Table 5**). Two real-time PCR assays were subsequently used in order to confirm the MLPA results with primers located in exon 1 and exon 2 of *CYP1B1*. The results of qPCR were 100% in agreement with those of MLPA data obtained (**Figure 3A**). Furthermore, no *CYP1B1* deletion alleles were detected among the studied subjects.

For *CYP2C9*, heterozygous deletion was detected in studied subjects by MLPA with copy number probes designed for exons 1, 7, 8(1), 8(2), and 9. In total of 154 subjects, 2 genotypes were observed with frequencies that varied from 1.95% (Wt/Del) to 98.05% (Wt/Wt) (**Table 4**). Two identified *CYP2C9* alleles were Wt (1 copy) and Del (0 copy) accounting for 99% and 1%, respectively (**Table 5**). Two real-time PCR assays were used to confirm the MLPA results with primers located in exon 4 and exon 7 of *CYP2C9*. Both exons 4 and 7 of the gene showed the loss of *CYP2C9* copy number, indicating the accuracy of MLPA (**Figure 3B**). Additionally, *CYP2C9* duplication alleles were not determined in this study.

For *CYP2E1*, heterozygous duplications were detected in 3/154 subjects (1.95%) by MLPA with copy number probes designed for exons 5, 6, and 8. Additionally, heterozygous deletions were found in only 1/154 subjects (0.65%) (**Table 4**). Three observed *CYP2E1* alleles consisted of Dup (2 copies), Wt (1 copy) and Del (0 copy), which made up 1%, 98.7%, and 0.3%, respectively (**Table 5).** Two real-time PCR assays were applied in order to verify the MLPA results using primers located in exon 8 and exon 9. Both exons of *CYP2E1* showed the gain of copy number in 2 samples tested compared with reference sample. The loss of *CYP2E1* copy number in tested samples was also in agreement with the data observed in MLPA (**Figure 3C**). Besides, no *CYP2E1* deletion carriers were identified in this study.

### Comparison of *CYP450* CNVs between Vietnamese Kinh and global populations

We further compared the CNVs observed in *CYP2A6* and *CYP2E1* between Kinh Vietnamese and populations from different geographical areas in the world (**Table 6**). For *CYP2A6*, no significant difference in *CYP2A6*Dup allele (0%) was observed between the present study and other populations, except for Ashkenazi Jews (*P* < 0.05). In Kinh Vietnamese, frequency of *CYP2A6*Del was significantly higher than those found in other populations, including Asian. For *CYP2E1*, frequency of *CYP2E1*Dup was significantly lower compared with data observed in African-American and Hispanic. Both *CYP2A6*Wt and *CYP2E1*Wt allele frequency detected in the studied subjects were similar to those previously figured out in other populations.

**Table 6.** Comparison of *CYP450* CNVs frequency between Kinh Vietnamese and worldwide populations

**Population**	**N**	**Alleles/copy number of *CYP2A6***			**References**

		**Dup (2)**	**Wt (1)**	**Del (0)**	
***CYP2A6* copy number alleles frequency**					
Kinh Vietnamese	154	ND	0.89	0.11	Present study
Chinese	96	-	0.849	0.151	[9]
Finns	100	-	0.96	0.001^***^	
Spaniards	100	-	0.965	0.005^***^	
Korean	209	-	0.885	0.11	[10]
Japanese	92	-	0.799	0.201^**^	[11]
African-American	105	0.01	0.952	0.038^**^	[2]
Asian	102	0.015	0.902	0.083^**^	
Caucasian	103	ND	0.985	0.015^***^	
Hispanic	109	ND	0.986	0.014^***^	
Ashkenazi Jews	123	0.02^*^	0.98	ND^***^	
Colombian	123	0.004	0.976	0.02^***^	[12]

**Population**	**N**	**Alleles/copy number of *CYP2E1***			**References**

		**Dup (2)**	**Wt (1)**	**Del (0)**	
***CYP2E1* copy number alleles frequency**					
Kinh Vietnamese	154	0.01	0.987	0.003	Present study
African-American	105	0.038^*^	0.962	ND	[2]
Asian	102	ND	1	ND	
Caucasian	103	0.029	0.971	ND	
Hispanic	109	0.041^*^	0.959	ND	
Ashkenazi Jews	123	0.004	0.996	ND	
Colombian	123	0.016	0.984	ND	[12]

* *P* < 0.05.

** *P* < 0.01.

*** *P* < 0.001.

CNVs, copy number variations; N, number of subjects; ND, not detected.

## Discussion

Of the 11 studied *CYP450* genes, the gene with the highest CNV frequency observed was *CYP2A6,* including both duplication and deletion alleles. As previous works described, *CYP2D6* together with *CYP2A6* are genes encoded for Phase I drug metabolism enzyme, which displayed the greatest number of CNVs [2, 5]. The most well-known CNV genes among *CYP450* are *CYP2D6*; the clinical impact of CNVs in this gene on a wide range of drugs have been reported in numerous studies. In fact, patients with duplicated *CYP2D6* presented with low-serum concentration of paroxetine for depression treatment [13] or lower plasma concentration of donepezil while no clinical improvement observed in Alzheimer's treatment [14]. For using painkillers, *CYP2D6* duplicated allele increase enzyme expression and activity, which is responsible for opioids poisoning [15]. At the moment of preparing the manuscript, *CYP2D6* variants including the CNVs in Kinh Vietnamese were already published and we thereby further discuss other *CYP450* genes.

In this study, *CYP2A6*Dup made up 1.16%, which is similar to that detected in the Asian and other populations in the world, except for Ashkenazi Jews. Notably, *CYP2A-6*Del was found at a much higher frequency (11%) in Kinh Vietnamese, which is also comparable with other populations in Asia such as the Chinese [9] and Korean [10]. Meanwhile, the Japanese showed a significantly higher prevalence of *CYP2A6*Del compared with the data of the current study [11]. An earlier research showed a significantly lower frequency of *CYP2A6*Del in Asians [2]. However, as the exact ethnic origins of the enrolled subjects were not mentioned in the study, this difference was possibly due to the selected subjects having come from various geographical areas in Asia [2]. Owing to the divergence of *CYP2A6*Dup and *CYP2A6*Del frequency in Asia, it has been suggested that different pressure of natural selection likely acted on certain allele [2]. Although deleterious variants usually underwent purifying selection [16], the high frequency of *CYP2A6*Del in Asia could be explained as the protective effect of this defective allele against lung and head and neck cancers in Asian populations [17]. *CYP2A6* is well known as the primary metabolizing enzyme of nicotine, and in turn has an influence on smoking behavior. Furthermore, this enzyme also metabolizes several clinically relevant substrates, of which some of the most relevant are coumarin, tegafur, letrozole, valproic acid, and pilocarpine. Most studies assess the clinical impact of *CYP2A6*Del on the metabolism of these substrates, yet the obtained results were still limited. For tegafur, a finding in Japanese cancer patients indicated that individuals having *CYP2A6*Del allele could experience less exposure to the active metabolite of tegafur-5-fluorouracil [18]. Similarly, the *in vitro* formation of 5-fluorouracil in liver chromosomes was decreased in donors carrying *CYP2A6*Del compared with donors without this variant [21] [19]. On the contrary, it remains to be seen whether *CYP2A6* variations have a significant effect on other substrates (valproic acid, pilocarpine), metabolism, as well as treatment outcome. There are currently no *CYP2A6* CNVs included in the drug label approved by the FDA as well as guidelines from the Clinical Pharmacogenomics Implementation Consortium. Nevertheless, the common *CYP2A6*Del frequency in Kinh Vietnamese and other Asian populations should be taken into account.

For *CYP1B1* and *CYP2C9*, little is known about the CNVs of these genes among different ethnic populations in the world. In this work, *CYP1B1*Dup and *CYP2C9*Del were both detected at only 1%. In 2019, a study analyzing CNVs of *CYP450* in the Colombian population showed that *CYP-2C9*Del allele made up 0.4% while no *CYP1B1*Dup was observed in 123 studied subjects [12]. For *CYP2E1*, to our best knowledge, there were only 2 reports on CNVs of this gene, in which no *CYP2E1Del* was identified in Colombians as well as in representatives of 5 different populations (African-American, Asian, Caucasian, Hispanic and Ashkenazi Jews) [2, 12]. A recent work focusing on *CYP2C* locus structural variants from almost 100,000 individuals also identified low CNVs frequency of pharmacogenes [20]. Taken together, these data demonstrated that CNVs in other *CYP450* genes are less common in all populations compared with *CYP2A6* and *CYP2D6*. Until now, the Pharmacogene Variation Consortium (pharmvar.org) has reported a diverse number of variants of *CYP1B1* (26 in total), *CYP2C9* (62 in total), and *CYP2E1* (7 in total) and most of these were SNPs and indels. Thereby, the impact of CNVs of pharmacogenes *CYP1B1*, *CYP2C9,* and *CYP2E1* on drug response is possibly less significant than the SNPs of these genes. However, it should be noticed that individuals who carry *CYP450* deletion/duplication could likely be at risk of insufficient drug response/drug toxicity due to poor metabolizer/ultra-rapid metabolizer phenotype. Although the translation of pharmacogenes CNVs into clinical intervention remains questionable, the clinical relevance of uncommon CNVs in these genes still should not be ignored.

In this study, 4 out of 10 of the studied genes showed the CNVs by MLPA technique. The accuracy of this method in identifying the genetic CNVs was confirmed by additional methods such as PCR and qPCR. However, the limitation of this study lies in lacking the evidence of breakpoint regions for each CNV detected in *CYP1B1* (duplication), *CYP2E1* (deletion and duplication), *CYP2C9* (deletion), and *CYP2A6* (deletion). This is due to the limited number of probes designed for only selected exonic regions of *CYP450* genes. In fact, the structure of locus harboring *CYP2E1* duplication or *CY2A6* deletion was discovered. Currently, there are no reports on the exact junction regions of *CYP1B1* duplication, *CYP2C9* deletion, and *CYP2E1* deletion. Depending on these junctions, whole exome sequencing or high-resolution method such as array Comparative Genomic Hybridization combined with Sanger sequencing might further resolve this hurdle of our research. Such worthy works would provide comprehensive evidence regarding the genetic architecture of pharmacogenes with structural variations.

## Conclusion

This is a comprehensive study in Vietnam that describes the CNV profile of *CYP450* genes in a cohort of the Kinh population. Alongside *CYP2D6*, our data further revealed the existence of CNVs in 4 genes including *CYP1B1*, *CYP2A6*, *CYP2C9,* and *CYP2E1*. This research supports the essential knowledge of *CYP450* CNVs prevalence in Vietnam, whose possible impact on physical health as well as drug metabolism should be further clarified.
